# Resolution of Stubborn Monkeypox With Tecovirimat in an HIV Patient

**DOI:** 10.7759/cureus.63407

**Published:** 2024-06-28

**Authors:** Landen S Burstiner, Monica Rodriguez, Hui Jun Guo, Manali Desai, Avni Agrawal, Loruanma Lam, Jorge Verdecia

**Affiliations:** 1 Internal Medicine, University of Florida College of Medicine - Jacksonville, Jacksonville, USA; 2 College of Medicine, University of Florida College of Medicine, Gainesville, USA; 3 Neurology, University of Florida College of Medicine - Jacksonville, Jacksonville, USA; 4 Infectious Disease, University of Florida College of Medicine - Jacksonville, Jacksonville, USA

**Keywords:** rash, infectious disease, department of health, hiv, monkeypox, tecovirimat

## Abstract

A 40-year-old male with a history of human immunodeficiency virus (HIV) (CD4 absolute count 57 cells/uL) presented to the Emergency Department complaining of large, swollen abscesses on his face, right hand, and feet. He reported the outbreak of the lesions occurred four months ago and coincided with a week-long episode of diarrhea, rectal pain, and perirectal and inguinal lymphadenopathy. Physical exam was significant for a full-thickness fluid collection on the sole of the right foot, a plantar abscess on the left foot, an open, crusted ulcer on the left fifth finger, and several large, crusted lesions on the face. Of note, the patient was seen at a nearby hospital three months prior, underwent a biopsy that showed non-variola orthopoxvirus DNA via real-time polymerase chain reaction (PCR), and was diagnosed with monkeypox at that time. He was advised to pick up tecovirimat treatment from the Department of Health but stated it was unavailable when he arrived and never took it. On this admission, the lesion was again biopsied and detected non-variola orthopoxvirus DNA by real-time PCR. The patient was discharged on 600 mg tecovirimat orally twice daily for 14 days. At the 14-day follow-up, the patient's lesions had completely fallen off and were no longer painful.

## Introduction

The monkeypox virus is a zoonotic virus related to the smallpox virus (both members of the genus *Orthopoxvirus*). Before the outbreak in March of 2022, monkeypox reports had been limited to countries where it was endemic [1]. The typical presentation of monkeypox includes an episode of prodromal symptoms followed by a characteristic vesiculopustular rash [2]. Most cases have 10 or fewer lesions, and lesions do not typically persist past four weeks in immunocompetent individuals [2]. Thornhill et al. published a case series of 528 infections, in which 41% of monkeypox cases also had an underlying human immunodeficiency virus (HIV) infection [1]. Among individuals with HIV with a low viral load, high CD4 count, and antiretroviral therapy (ART), the clinical course of monkeypox does not appear to be significantly different than it would be for those without HIV [1,3]. However, in individuals with poorly controlled or previously unknown HIV infection, the disease course has been described to be worse and can involve more lesions and complications [4-8]. Tecovirimat, a viral protein p37 inhibitor, acts by disrupting envelope formation in orthopoxviruses and has shown promise as a treatment for monkeypox [9]. Viguier et al. described one such case in August 2022 of an individual with advanced HIV, whose symptoms resolved and lesions improved after 14 days on tecovirimat [5].

Monkeypox lesions are considered pathognomonic and can be pustular papules with a central umbilicated dip, fluid-filled vesicles, ulcerations, or eschars [10]. The characteristic monkeypox rash begins with pruritic or painful macular lesions, which progress to papules by day 3, vesicles on days 4 and 5, pustules on days 6 and 7, and scabbing on days 7 through 14 [11].

## Case presentation

**Figure 1:**
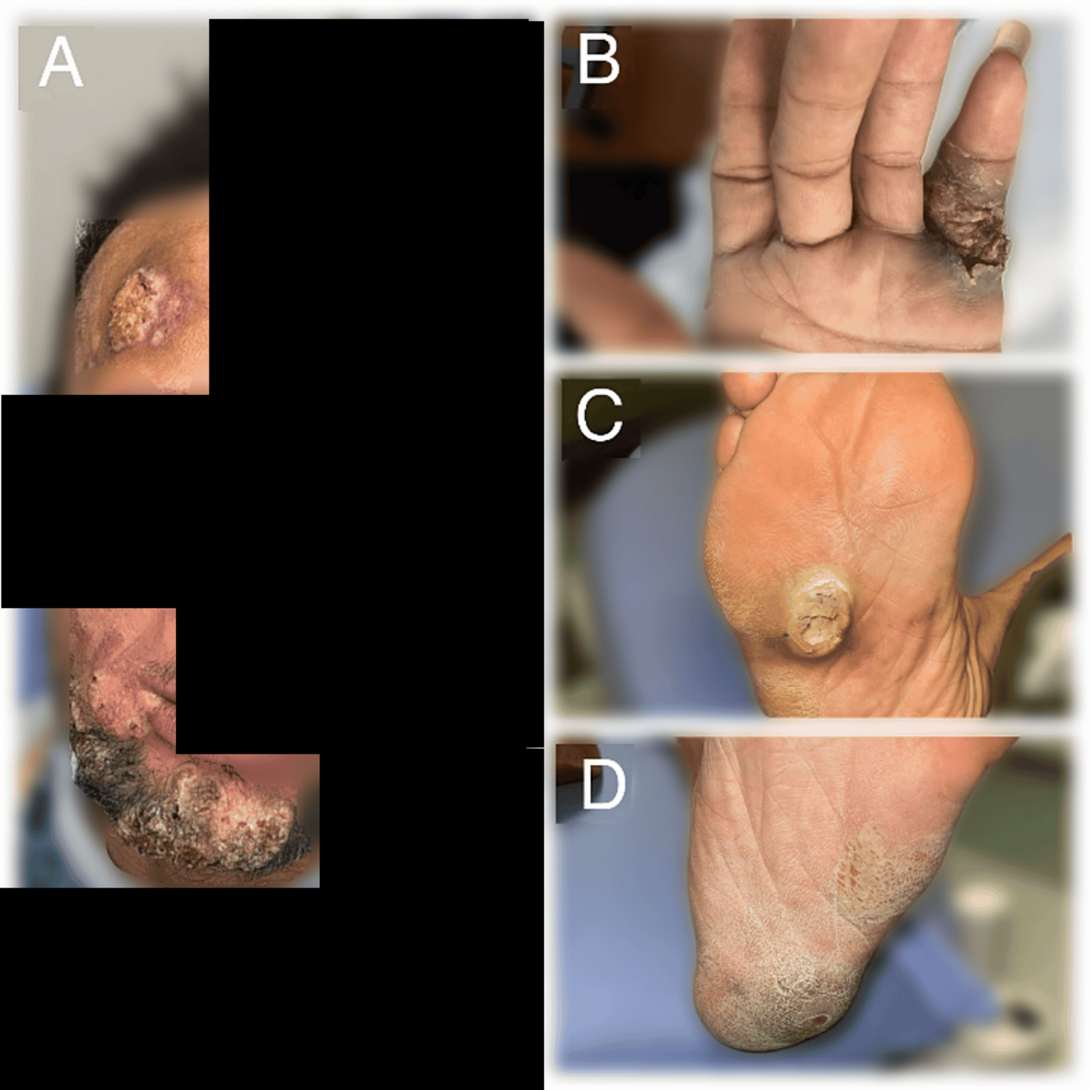
A) Crusted lesions on the chin and right forehead; B) Open, crusted ulcer on the left fifth finger; C) Plantar abscess on the right foot; D) Full-thickness fluid collection on the left foot

**Figure 2:**
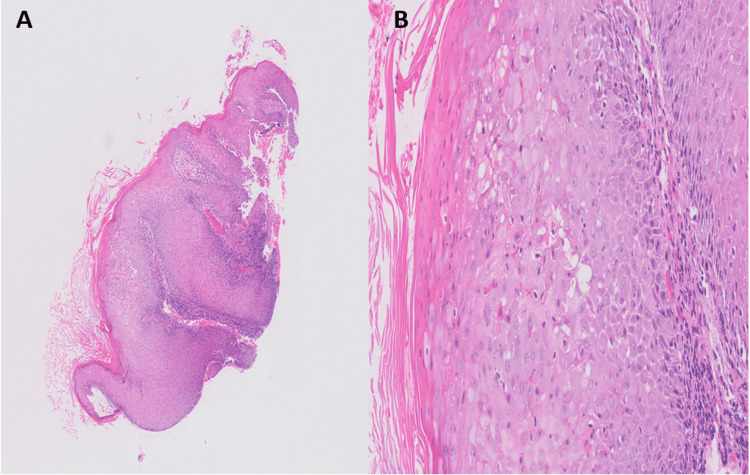
A) Skin biopsy of the erythematous rash at low power (x2) showing acanthosis; B) High power picture (x20) showing the epidermis with keratinocytes ballooning and focal eosinophilic ground glass appearance of the keratinocytes

A 40-year-old male with a history of HIV (unknown adherence with ART) presented to the Emergency Department complaining of large swollen abscesses on his face and the soles of his feet, which were causing severe pain while ambulating. He also noted a painful lesion on his right hand. He reported the outbreak of the lesions coincided with a week-long episode of diarrhea, rectal pain, and perirectal and inguinal lymphadenopathy about four months ago, and the lesions had been gradually progressing in size since. On presentation, the patient was normotensive, eupneic, and afebrile with sinus rhythm. Physical exam revealed a full-thickness fluid collection on the sole of the right foot, a plantar abscess on the left foot, an open, crusted ulcer on the left fifth finger, several large crusted lesions on the chin, and a large, crusted lesion on the right side of the forehead (Figure 1). Herpes simplex virus (HSV) polymerase chain reaction (PCR) for both HSV-1 and HSV-2 from the left finger ulcer was negative. HIV-1 RNA PCR showed a viral load of 40,100 copies/mL. The patient's CD4 absolute count was 57 cells/uL (reference range 260-1557), and C-reactive protein was elevated at 12.80 mg/L (reference range <8.0). X-rays of the right and left foot showed soft tissue swelling at the fifth metatarsophalangeal joint but were negative for acute osseous abnormalities. A punch biopsy of the lesion on the right forehead was sent for pathology, which showed acanthosis and serum crust with inflammatory cells (Figure 2). The HSV, periodic acid-Schiff (PAS), and Grocott's methenamine silver (GMS) stains were negative. The pathologist read these findings as non-specific and stated that no malignancy was seen. No viral inclusions were present.

**Figure 3:**
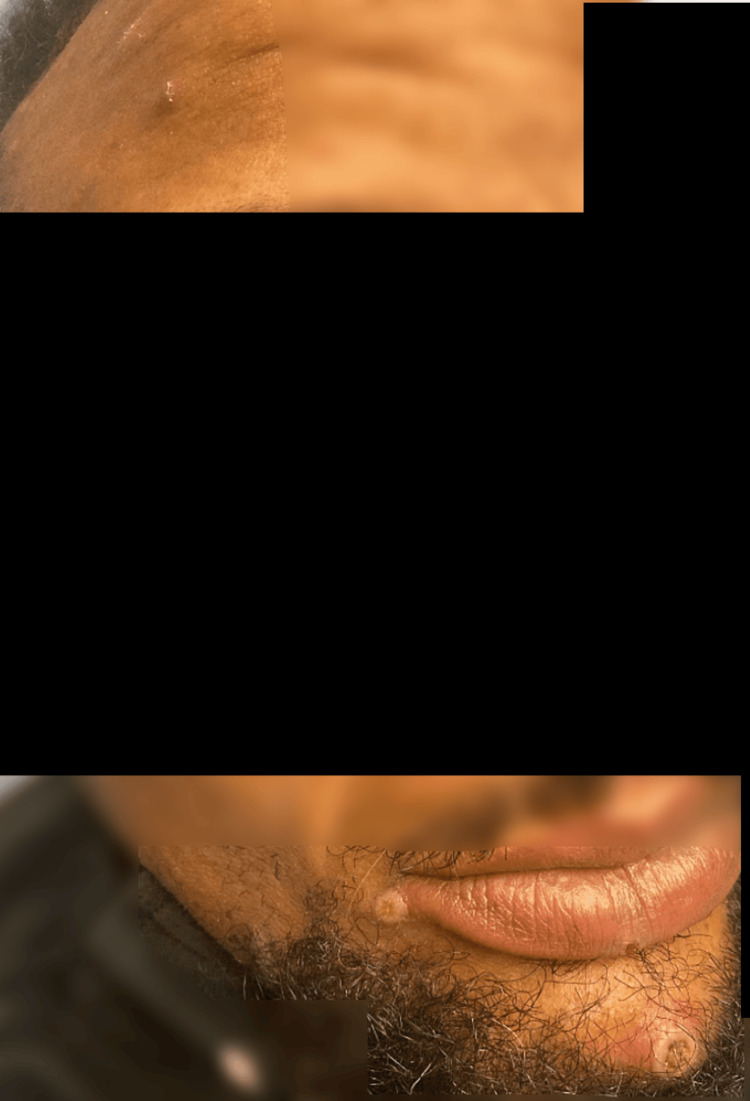
Multiple small umbilicated papules on the chin, right side of the forehead

Of note, this was actually the patient’s second presentation. He was seen at a nearby hospital three months prior and was diagnosed with biopsy-proven monkeypox. On his initial presentation, he complained of diarrhea, rectal pain, perirectal and inguinal lymphadenopathy, and two weeks of umbilicated rectal and facial lesions (Figure 3), and underwent lesion biopsy which detected non-variola orthopoxvirus DNA via real-time PCR. CD4 absolute count was 30 cells/uL, and HIV-1 PCR was 83,800 copies/mL at that time. He was advised to pick up tecovirimat treatment from the Department of Health, but he stated that it was unavailable when he arrived and never took it. The Health Department was contacted and confirmed tecovirimat had never been dispensed to the patient.

**Figure 4:**
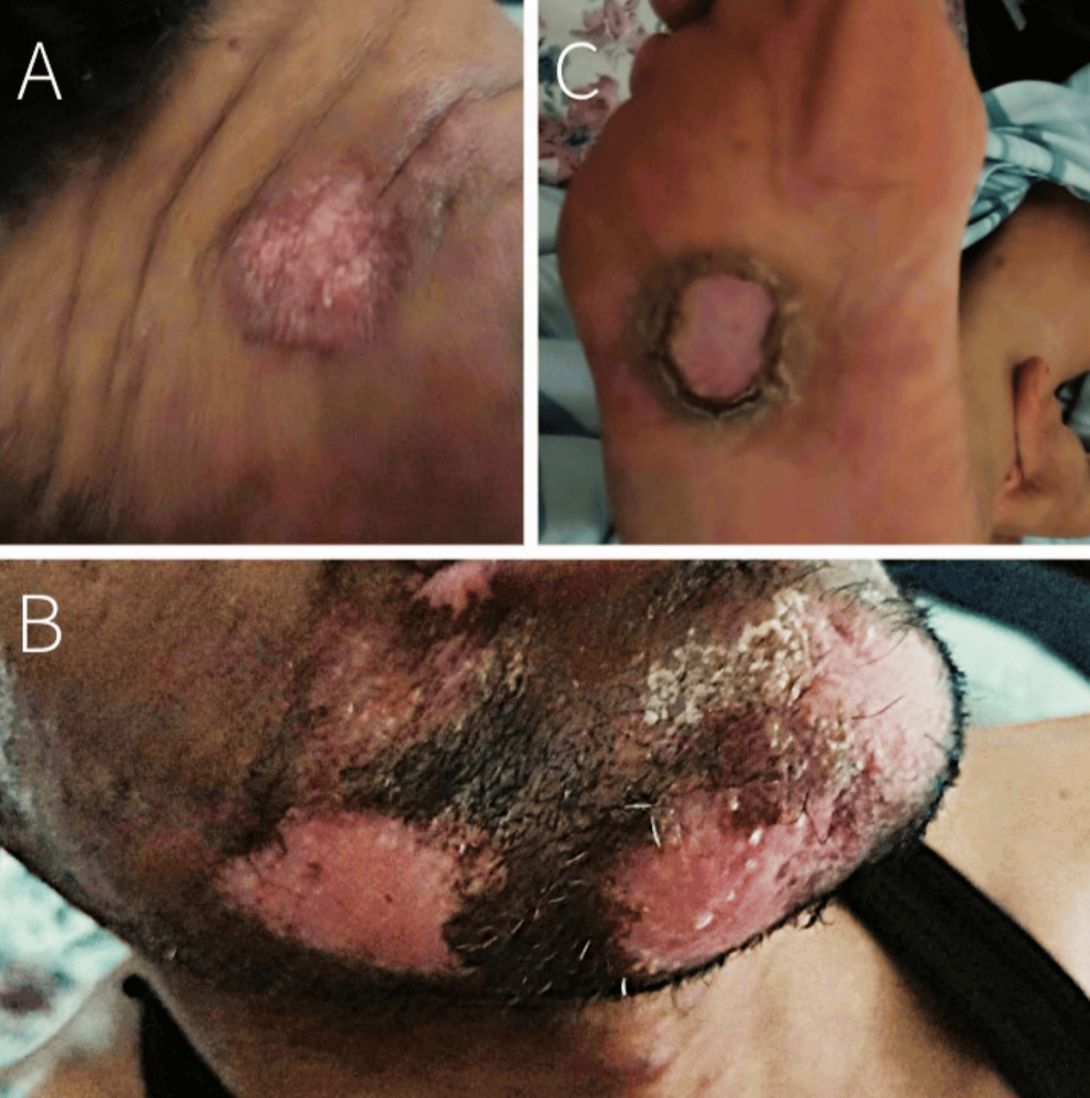
A) Right forehead lesion; B) Chin lesions; C) Right foot lesion

On this admission, the right forehead lesion was again biopsied and sent to an external laboratory, which detected non-variola orthopoxvirus DNA by real-time PCR. With this admission, we coordinated with the Department of Health to ensure tecovirimat was available to the patient. He was discharged on 200 mg tecovirimat capsules with instructions to take three capsules twice daily (1200 mg total per day) for 14 days. At the 14-day follow-up, the patient's lesions had completely "fallen off" and were no longer painful (Figure 4).

## Discussion

In the emerging field of monkeypox, it is crucial to evaluate the efficacy of various potential therapies, as none have been FDA-approved. Our patient was able to receive tecovirimat due to a non-research expanded access investigational new drug (EA-IND) protocol (sometimes called "compassionate use") that allows for the use of tecovirimat for primary or early empiric treatment of non-variola orthopoxvirus infections [9]. There are currently two clinical trials evaluating tecovirimat in treating monkeypox: a randomized controlled trial started in September 2022 based in America [12], and a separate trial in the Democratic Republic of the Congo [13]. These trials may provide the necessary findings to validate or refute the use of tecovirimat in monkeypox.

Treatment of immunocompromised patients with monkeypox is of particular importance, as these patients are at risk of harboring long-term monkeypox infection, as opposed to most immunocompetent patients who appear to resolve monkeypox infections within four weeks without requiring intervention [2]. Other potential treatments for monkeypox include brincidofovir, which has shown promise in animal models but was not tolerated in a three-patient case series due to elevated liver enzymes [14]. There are two vaccinations currently in use for monkeypox: JYNNEOS, which is FDA-approved for monkeypox in patients ages 18 years and older, and ACAM2000 approved for the prevention of monkeypox via an EA-IND application [14].

## Conclusions

Our immunosuppressed patient experienced discomfort and pain for more than three months due to documented monkeypox lesions, with no signs of improvement. Following a course of tecovirimat, he had complete resolution of his monkeypox lesions with no reported side effects. Given the current absence of peer-reviewed medication trials, we recommend providers consider treating future immunosuppressed patients with non-abating, biopsy-proven monkeypox with tecovirimat until trials are completed and state otherwise.
